# The neuroactive potential of the elderly human gut microbiome is associated with mental health status

**DOI:** 10.1371/journal.pone.0343493

**Published:** 2026-04-15

**Authors:** Paulina Calderón-Romero, Benjamin Valderrama, Thomaz F. S. Bastiaanssen, Patricia Lillo, Daniela Thumala-Dockendorff, Gerard Clarke, John F. Cryan, Andrea Slachevsky, Christian González-Billault, Felipe A. Court

**Affiliations:** 1 Center for Aging Research and Healthy Longevity, Faculty of Sciences, Universidad Mayor, Santiago, Chile; 2 Fondap Geroscience Centre for Brain Health and Metabolism, Santiago, Chile; 3 APC Microbiome Ireland, University College Cork, Cork, Ireland,; 4 Department of Anatomy and Neuroscience, University College Cork, Cork, Ireland; 5 Department of Psychiatry, Amsterdam University Medical Centers Location Vrije Universiteit Amsterdam, The Netherlands; 6 Department of Neuroscience and South Neurologic Department, Faculty of Medicine, University of Chile, Santiago, Chile; 7 Psychology Department, Faculty of Social Sciences, Universidad de Chile, Santiago, Chile; 8 Department of Psychiatry and Neurobehavioural Science, University College Cork, Cork, Ireland; 9 Neuropsychology and Clinical Neuroscience Laboratory (LANNEC), Interdisciplinary Nucleus for Physiology, Biophysics and Pathophysiology– Institute of Biomedical Science, Neuroscience and East Neuroscience Departments, Faculty of Medicine, University of Chile, Santiago, Chile; 10 Neurology and Psychiatry Department, Clínica Alemana-University Desarrollo, Santiago, Chile; 11 Memory and Neuropsychiatric Center (CMYN), Memory Unit - Neurology Department, Hospital del Salvador and Faculty of Medicine, University of Chile, Santiago, Chile; 12 Department of Biology, Faculty of Sciences, Universidad de Chile, Santiago, Chile; 13 Department of Neurosciences, Faculty of Medicine, Universidad de Chile, Santiago, Chile; 14 Public Nutrition Unit, Institute for Nutrition and Food Technology, Universidad de Chile, Santiago, Chile; 15 Buck Institute for Research on Aging, Novato, California, United States of America; 16 Centro Científico y Tecnológico de Excelencia Ciencia & Vida, Fundación Ciencia & Vida, Santiago, Chile; Monash University Malaysia, MALAYSIA

## Abstract

Ageing is usually associated with physiological decline, increased mental health issues, and cognitive. deterioration, alongside specific changes in the gut microbiome. However, the relationship between the neuroactive potential of the gut microbiome and mental health and cognition among the elderly remains less explored. This study examines a cohort of 153 older Chilean adults with cognitive complaints, assessing anthropometric data, mental health via five distinct tests, and gut microbiome composition through 16SV4 sequencing. Our findings reveal associations between anthropometric factors and depression scores in mental tests of participants with their gut microbiome composition. Notably, depression was associated with changes in the abundance of *Lachnospiraceae Eubacterium xylanophilum group* and *Fusobacteriaceae Fusobacterium*. Additionally, bacterial pathways involved in metabolising neuroactive compounds such as tryptophan, short-chain fatty acids, p-cresol, glutamate, and nitric oxide were associated with participant age, sex, and cognitive performance. Moreover, participants’ sex was associated with the neuroactive potential of specific bacteria, suggesting a role of the gut microbiome in sex-related mental health differences in the elderly. Altogether, this study provides the first evidence from a South American cohort linking the inferred neuroactive potential of the gut microbiome to cognitive and psychological function in older adults with cognitive complaints, offering novel insights into microbiota-based mechanisms that may contribute to mental health and ageing.

## 1. Introduction

According to global projections, the percentage of people over 60 years old will nearly double by 2050 [1]. As the global population ages, health concerns related to ageing are becoming increasingly important. Among the conditions affecting older adults, physical diseases, as well as cognitive disorders such as dementia and depression are the most common [2–4]. Ageing is a natural and complex process characterised by a steady decline in mental sharpness and independence in daily activities, the extent of which can be determinant for ageing well and healthy [5,6]. However, the mechanisms underlying the decline and the factors driving its individual-specific differences in the rate of decline remain to be fully elucidated.

Within the spectrum spanning from healthy ageing to dementia, cognitive complaints defined as self- or hetero-reported experiences of memory loss or cognitive decline—have emerged as a significant risk factor for future cognitive impairment, functional decline, and progression to dementia [7]. These complaints have been linked to varying degrees of cognitive and functional impairment, as well as the presence of neuropsychiatric symptoms [8]. Identifying individuals within the community who report cognitive disturbances and are at higher risk for functional decline and dementia conversion could pave the way for more personalised prevention strategies tailored to this high-risk group [9]. In this context, specific mental tests can be utilised to assess changes in brain function during ageing and to stratify risk groups. Recent improvements in test sensitivity and specificity have increased their accuracy in assessing cognitive issues in older individuals. Several tests have been developed and validated to cover different aspects of mental health in older people, including tests such as Activities of Daily Living Questionnaire (ADLQ) [10], Everyday Cognition (ECog) [11], Alzheimer Disease 8 (AD8) [12,13], Cognitive Reserve Scale (CRS) [14] and the Geriatric Depression Scale (GDS) test [15,16]. These provide physicians with valuable information for classifying cognitive level, memory loss, and other cognitive complaints that could affect daily life.

Along with well-established potential decline in brain functions and mental health, new studies show evidence supporting alterations in the gut microbiota of people transitioning from adulthood to elderly. These changes are characterised by a loss of overall microbial diversity and a greater inter-individual variation, also referred to as a higher degree of gut microbiome uniqueness [17,18]. Additionally, evidence suggests ageing is characterised by a decrease in Firmicutes and an increase in Bacteroidetes phyla [19], and that longevity has been associated with an increased abundance of *Clostridium cluster XIV*, Ruminococcaceae, *Akkermansia* and Christensenellaceae, known to produce molecules that may modulate human inflammation [20–24]. Moreover, it’s been suggested that age-related changes in the gut microbiome can accelerate the progression of ageing and inflammation, impacting overall well-being [25]. Therefore, changes in the gut microbiome have recently gained interest as factors that could distinguish between healthy and unhealthy ageing in the human population. Indeed, research has suggested a role for the gut microbiome as a potential mediator of overall health in older populations [17,26–28].

Interestingly, the gut microbiome has also been described as a modulator of the bidirectional communication between the brain and the gut, leading to the coining of the term microbiome-gut-brain axis [29]. Evidence suggests that gut bacteria can produce neuroactive metabolites [30,31], such as short chain fatty acids (SCFAs; acetate, propionate, butyrate) [32], which may modulate neuronal inflammation and has been linked to depression status [33]. Therefore, targeting the gut microbiome could lead to an improvement in mental health of the elderly, as depression is a common feature. Indeed, patients with major depressive disorder experience a reduction in SCFAs, while supplementation with butyrate has shown antidepressant effects in animal studies [34,35]. Moreover, depression is often associated with a loss of microbial stability in patients, which has been hypothesised to exacerbate depressive symptoms [36]. On the other hand, the production of bile acids and neurotransmitters may lessen the severity of depression [37]. Bacterial genera, such as *Enterobacter* and *Burkholderia* have shown a positive correlation with depressive symptoms and negative correlations with brain structures responsible for memory and emotional regulation [38].

In addition to SCFAs, other gut bacteria-derived metabolites may interact with the vagus nerve, the main direct route of communication between the central and peripheral nervous systems [39]. Therefore, the microbiota-gut-brain axis plays crucial roles in various processes impacting human mental health, including neurodevelopment, psychological and psychiatric behaviours, age-associated disorders, and neurodegenerative diseases [40,41]. However, whether the gut microbiome plays a role in modulating the mental health of the elderly, or its involvement in the decline in mental capabilities is not fully understood.

In this context, our study provides insights on how the composition and neuroactive potential of the gut microbiome are linked to cognitive performance, depressive symptoms, and other physiological factors. This study explores the relationship between the inferred neuroactive potential of the human gut microbiome and cognitive performance in older adults with cognitive dysfunction. Therefore, our findings offer new perspectives on the role of the gut microbiota as a possible modulator of mental health in this population and represent an initial exploration of the potential of the gut microbiome as a target for therapeutic strategies in ageing. Therefore, this study aimed to characterize the composition and inferred neuroactive potential of the gut microbiome and explore their associations with cognitive performance, depressive symptoms, and other functional and anthropometric factors in a cohort of older Chilean adults with cognitive complaints. Rather than comparing against a control group, the purpose of this work was to describe this population in detail and identify internal associations that may reflect early microbiome signatures related to cognitive and mental health outcomes.

Considering the descriptive and observational nature of this study, we hypothesized that variations in cognitive and psychological performance would be associated with distinct taxonomic and functional microbiome profiles, particularly in pathways involved in the synthesis and degradation of neuroactive compounds. Therefore, our findings offer new insights into the role of the gut microbiota as a modulator of mental health of this population and represents an initial exploration on the potential of the gut microbiome as a target for therapeutic interventions in the context of mental health in the elderly.

## 2. Methods

### 2.1 Cohort

The population of 153 individuals described in this study corresponds to the baseline assessment of the Chilean GERO cohort. A community-based cohort aimed at analysing predictors of functional decline and dementia progression in elderly individuals with cognitive complaints residing in the community as previously described [9]. Cognitive complaints were defined as subjective concerns about memory or cognitive function, either self-reported or reported by a knowledgeable informant. In addition, all participants were required to have a Clinical Dementia Rating—Frontotemporal Lobar Degeneration (CDR-FTLD) score ≤ 0.5. This is a clinical project of the Geroscience Center for Brain Health and Metabolism. Cohort participants were recruited from the general population using a door-to-door strategy between November 2017 and December 2021 in Santiago, Chile. Participants were required to be 70 years or older with cognitive complaints reported by themselves or a reliable informant. Additionally, participants needed to have a Clinical Dementia Rating Scale (CDR-FTLD) score equal to or less than 0.5, live in the community, and have a knowledgeable informant. Exclusion criteria included diagnosed dementia or screening tests indicating dementia, cognitive impairment (Mini-Mental State Examination (MMSE) scores < 21) [42], and functional impairment (Pfeffer questionnaire score > 2) [43]. Additionally, participants were excluded if they had a history of institutionalisation, illiteracy, severe sensory or mobility impairments, major psychiatric or neurological disorder (such as schizophrenia, brain tumour, subdural hematoma, progressive supranuclear palsy, head trauma, or recent stroke), or a fatal with a prognosis of less than one year. Although the original protocol allowed the inclusion of individuals with a history of stroke older than three months, no participants with prior stroke were enrolled in the cohort. In addition, any incident strokes reported during follow-up were documented and led to participant removal from the cohort. Accordingly, no participants with stroke were included in the baseline analyses presented in this study. Medication use and comorbidities were systematically recorded for all participants, including dosage information. For the present study, recent antibiotic use (within the previous month) and history of major gastrointestinal surgery were assessed as exclusion criteria, and no participants met these conditions. Among the registered comorbidities, depressive symptoms were specifically examined due to their relevance to gut microbiome composition. All study participants had a reliable informant who provided information about their functional abilities. After inclusion, all subjects completed an extensive neurological, neuropsychological, and neuroimaging evaluation (see Slachevsky, et al., 2020 [9] or a detailed description of inclusion and exclusion criteria, and of the general protocol). Each participant provided signed informed consent, and the study was approved by the Ethic Committee of the Servicio de Salud Metropolitano Oriente, Santiago (Chile) (approval references: SEC No. 1140423, May 5, 2015, and follow-up approval, September 20, 2016). The samples were collected between October 2018 and January 2021. The authors accessed the data on 8 February 2023 and had no access to any personal identifiers that could individualize participants. The project is funded by the Chilean National Agency of Research (ANID, FONDAP 15150012).

### 2.2 Data collection

Anthropometric measurements, blood tests, and stool collection, as well as neuropsychological assessments involving cognitive, functional, and neuropsychiatric tests, were conducted [9]. The results of these questionnaires were collected concurrently with the biological samples, ensuring that the data from both sources were gathered during the same period. Anthropometric data included age, sex and BMI. Cognitive status was assessed using the Montreal Cognitive Assessment (MoCA) and the Addenbrooke’s Cognitive Examination III (ACE-III), covering five cognitive domains [44]. The MoCA score ranges from 0 to 30, with higher scores indicating better cognitive performance, while the ACE-III scores range from 0 to 100. Exclusion criteria include psychiatric or neurological disorders, with severe depression participants not being part of the cohort. However, depressive symptoms were assessed using the Geriatric Depression Scale (GDS), with scores ranging from 0 to 15 [42,43,45–47]. Informant-rated questionnaires, such as the Technology-Activities of Daily Living Questionnaire (T-ADLQ) were used to assess everyday functioning in three domains: Basic, Instrumental, and Advanced. The measurement range for each domain is from 0 to 100%, with higher percentages indicating greater functional impairment [10,48,49]. Additionally, the Everyday Cognition (ECog) questionnaire was used to measure cognitive abilities across six domains with a total of 39 questions, each scored from 1 to 5, with a higher total scores indicating more impairment [50]. The AD-8 questionnaire, consisting of 8 questions with a total score of 8, was used to evaluate functional changes typically associated with dementia [12,13]. Finally, the participation in cognitively stimulating activities across different life stages was assessed using the Cognitive Reserve Scale (CRS) test, with a scale from 0 to 15 [14,51]. Importantly, the depressive status of participants was determined through self-reporting according to the guidelines in the National Health Survey [52]. Cognitive and depressive symptom scores were analyzed as continuous variables, and no diagnostic cut-offs were applied. Sex-related differences were evaluated using independent-samples t-tests for continuous variables, while differences in sex proportions were assessed using the chi-square (χ²) test. To control for multiple comparisons, p values were adjusted using the Benjamini–Hochberg false discovery rate procedure.

### 2.3 Sample collection

Samples were obtained at the Memory and Neuropsychiatric Clinic (CMYN) Neurology Department, Hospital del Salvador and Faculty of Medicine, University of Chile, Santiago, Chile [9]. The stool samples were collected in OMNIgene gut tubes (DNAgenotek, OMR-200), and the participants performed the sample collection at home following the instructions provided by a nurse. These tubes contain a proprietary stabilizing buffer that preserves microbial DNA integrity at room temperature for up to 60 days, ensuring microbiome profile stability during transport and storage without the need for a cold chain. The tubes containing the samples were then retrieved and stored for no more than one month at room temperature until their arrival at the laboratory, where they were kept at −80 ºC until processing.

### 2.4 Sample processing

DNA extraction from the samples was carried out utilising the IHMS SOP 06 V1: STANDARD OPERATING PROTOCOL FOR FECAL SAMPLES (human-microbiome.org/index.php?id=Sop&num=006) and QIAamp Fast DNA Stool Mini Kit (Qiagen, 51604). The extraction process, as well as quality control, were conducted at the Center for Integrative Biology, Mayor University. A gut microbiome biobank was established and preserved at −80 ºC. The DNA samples were transported on dry ice for sequencing.

### 2.5 Sequencing

DNA sequencing was performed at the Alkek Center for Metagenomics and Microbiome Research (Department of Molecular Virology and Microbiology at Baylor College of Medicine, Houston, Texas, USA). The V4 region of the 16S rRNA gene was targeted using the primers 515F: GTGCCAGCMGCCGCGGTAA and 806R: GGACTACHVGGGTWTCTAAT [53]. The sequencing procedure involved performing 2x250 bp paired-end sequencing using an Illumina Miseq system in rapid run mode. Sequencing yielded a mean of 41,078 reads per sample with a standard deviation of 14,395. No sample had less than 10,000 reads after sequencing.

### 2.6 Bioinformatics and statistical analysis

#### 2.6.1 16S data analysis, taxonomic, functional annotation, and diversity within samples.

FastQ files with raw sequences were loaded into R (version 4.1.1), where the Divisive Amplicon Denoising Algorithm (DADA2) v. 1.22.0 [54] was used to construct a count table of amplicon sequence variants (ASV). The DADA2 workflow consisted of filtering and trimming low-quality reads, learning error rates, sample inference, merging paired reads, constructing an ASV table, removing chimeras and assigning taxonomy up to the genus level. For each step of the workflow default parameters were used with the exception of the function filterAndTrim from the dada2 R package. The following non-default values were used as arguments of the function: maxEE was set as c(2,5), truncQ as 2, trimLeft as 20 and truncLen as 200 to fix the length of the sequences used later in the analysis. After pre-processing, samples had a mean sequencing depth of 27,869 with a standard deviation of 11,478. No sample had less than 10,000 reads. Then, for taxonomy assignment, SILVA database (v 138) was used as a reference [55]. PICRUSt2 (version 2.4.1) [56] was used with its default parameters to infer the genomic content from 16S data, using the KEGG database [57] as a reference. The inferred genomic content was used to compute the abundance of Gut Brain Modules (GBMs), defined as a collection of 56 bacterial biochemical pathways involved in the synthesis and degradation of neuroactive molecules [30]. To determine the abundance of the GBMs, the R package omixer-rpm (version 0.3.3) [58] was used in stratified mode (i.e., determine GBMs for each independent bacterial genera), and in the unstratified mode (i.e., the combined profiles of every bacterium from a participant’s microbiome). In both analyses, default software parameters were used. For following analyses, the ASV table and the GBMs table were transformed using the Centered Log Ratios (CLR) transformation implemented in the vegan package (version 2.6.4) with a pseudocount of 2/3. Downstream statistical analysis was performed with R (version 4.2.0) and RStudio (version 2022.7.1.554).

#### 2.6.2 Microbiota community variation explained by metadata variables.

Variables included in our analyses were first checked to avoid high collinearity before statistical models were produced, for which a Pearson’s rho value higher than 0.7 was used as an exclusion cutoff. Variables in the metadata were scaled before being included in the models. First, distance-based redundancy analysis (dbRDA) was used to determine the contribution of variables in the metadata to the variations observed in the taxonomic composition of the gut bacteria among participants. This constrained ordination approach was selected over unconstrained methods such as PCoA or NMDS, as it explicitly incorporates explanatory variables. Dissimilarity was calculated using Aitchison’s distance (Euclidean distance on CLR-transformed data) as recommended for the analysis of compositional data [59], and dbRDA was performed using functions from the vegan package. For the analysis, variables previously found to influence the taxonomic composition of the gut microbiome were included in our statistical models. Therefore, we aimed to explain differences in the overall composition of the gut microbiome (i.e., the complete CLR-transformed bacterial counts matrix) using the following formula: “matrix ~ sex + age + bmi + self-reported depression + cognition + ADLQbasic + ADLQinstrumental + ADLQadvanced + batch”. The last variable, batch, was included to account for possible batch effects due to different sequencing runs. The same covariates were used to identify possible differences in the abundances of specific bacterial genera. To do so, Generalized Linear Models (GLMs) were fitted using the same formula used for the RDA analysis with the function fw_glm from the Tjazi R package (version 0.1.0), as recently recommended [60,61]. Calculated p-values were adjusted using the FDR Benjamini-Hochberg method. Adjusted p-values < 0.1 were deemed as statistically significant.

#### 2.6.3 Description of the neuroactive potential of the GERO cohort.

With the count tables of GBMs abundances and coverages, the prevalence of each GBM in the cohort was calculated as the percentage of the total of participants in which the GBM was detected. Importantly, GBMs were deemed present in the gut microbiome of a patient only if all the genes that were originally described as part of each GBM [30] were inferred to be present, in order to ensure a stringent detection criterion. Additionally, to assess if variables previously found to influence the taxonomic composition of the gut microbiome could also influence its neuroactive potential, a GLM for each GBM was fitted using the following formula “GBM_abundance ~ sex + age + bmi + GDS total score (ts) + ECog ts + ADLQbasic ts + ADLQinstrumental ts + ADLQadvanced ts + batch” as required by the function fw_glm from the Tjazi R package [60,61]. The same formula was used then to assess if those variables could also induce changes in the abundance of GBMs encoded in specific bacterial genera (i.e., using the stratified GBMs count table produced with omixerRpm software version 0.3.3 as explained previously). In both cases, the variable batch was included to account for possible batch effects due to different sequencing runs, and the calculated p-values were adjusted using the FDR Benjamini-Hochberg method. The adjusted p-values < 0.1 were deemed as statistically significant.

#### 2.6.4 Associations between participants’ scores in mental tests and their gut microbiome.

Scores achieved by participants in a battery of mental tests were used to test three hypotheses, namely: that (1) the taxonomic composition of the gut microbiome, or (2) the neuroactive potential of certain bacterial genera, or (3) the neuroactive potential of the entire gut microbiome are associated with the participant’s score in the mental questionnaires. To test these hypotheses, linear models were fitted using the scores of the participants in different mental tests as covariates explaining the abundance of either (1) a bacterial genus, (2) each GBMs found within each bacterial genus or (3) the abundance of each GBM in the entire gut microbiome. For the three hypotheses, the fw_glm function from the Tjazi package was used to fit GLMs, and the formula included the total scores (ts) of all the mental tests each participant answered as follows: “response ~ AD8 ts + MoCA ts + ECog ts + ACEIII ts + CRS ts + self-reported depression + batch”. The variable batch was included to account for possible batch effects due to different sequencing runs. Calculated p-values were adjusted using the FDR Benjamini-Hochberg method, and adjusted p-values < 0.1 were deemed as statistically significant.

## 3. Results

### 3.1 The gut microbiome composition of the GERO cohort is primarily influenced by anthropometric variables and depressive symptoms scores

**Fig 1:**
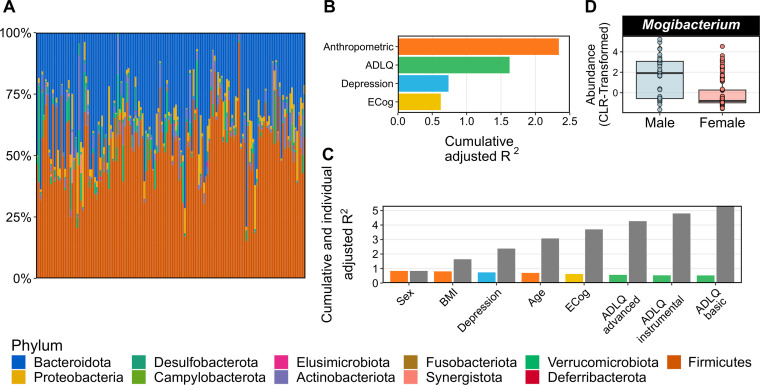
Taxonomic description of the microbiome and explanatory power of cohort covariates on the taxonomic composition of the gut microbiome of the GERO cohort. (A) The relative abundance of the microbiomes for 153 participants of the study. Each colour represents different phyla, as shown in the bottom legend. (B) The combined explanatory power of different cohort covariates pooled into 4 pre-defined categories: Anthropometric, ADLQ, Depression and Cognition. Each category is depicted with one colour. (C) The individual and cumulative explanatory power of each covariate included in the model used to explain changes in overall microbiome community variation. The colours in the bars correspond to the colours of the 4 categories used in panel B. Gray bars on the right of each coloured bar depict the cumulative effect of the covariates. Coloured bars are sorted in a decreasing order from left to right. (D) The genera *Mogibacterium* was found to be more abundant within male participants enrolled in the study. Benjamini-Hochberg method was used to adjust p-values after multiple comparisons. Statistical significance was determined with q < 0.1.

16S amplicon sequencing was used to characterise the taxonomic composition of the gut bacterial microbiome in the cohort. Results indicate that the microbiome of the GERO cohort is predominantly composed of the phyla Firmicutes (Mean = 53.7%, SD = 13.9%) and Bacteroidota (Mean = 34.1%, SD = 14.8%) (Fig 1A). Nine additional human-commensal phyla were detected in much lower abundance, including Proteobacteria, Verrucomicrobiota, Actinobacteriota, Synergistota, Desulfobacterota, Campylobacterota, Elusimicrobiota, Deferribacterota, and Fusobacteriota.

RDA analysis was conducted to disentangle the extent of the overall variation in the bacterial community that could be explained by different variables of the metadata. The anthropometric variables of the participants (Table 1, and S1 Table) accounted for the greatest amount of variation across the cohort (2.38%), followed by the scores obtained in the ADLQ test (1.56%), their depressive symptoms score according to the GDS (0.74%) and the scores in the ECog test (0.58%) (Fig 1B). We then studied the independent contribution of each metadata variable. According to the RDA, the three host-derived variables with the most explanatory potential were BMI (0.87%), sex (0.84%) and depressive symptoms score (0.74%), followed by the other anthropometric and cognitive variables, including the ADLQ scores, accounting for a total explained variance of 5.26% (Fig 1C).

**Table 1:** Anthropometric, cognitive and psychological characteristics of the 153 participants aggregated by sex.

Domain	Characteristics aggregated by sex	Female	Male	p-value	Score interpretation
Anthropometric	Number of participants	119 (77.77%)	34 (22.23%)	7.8 x 10^–11^	–
	Age	76.76 ± 5.13 (69–90)*	76.62 ± 4.69 (71–89)*	0.882	–
	Height	152 ± 7 (cm)	166 ± 7 (cm)	2.53 x 10^–14^	–
	Weight	66.56 ± 12.42 (kg)	78.31 ± 13.66 (kg)	1.8 x 10^−4^	–
	BMI	28.89 ± 5.28	28.16 ± 3.76	0.516	–
Cognitive	MOCA (0–30)	21.34 ± 4.92	21.47 ± 3.16	0.882	↓ Lower scores indicate poorer cognitive performance [43]
	ACE III (0–100)	78.55 ± 11.49	80.79 ± 9.96	0.240	↓ Lower scores indicate poorer cognitive performance [46]
	AD8 (0–8)	2.55 ± 1.78	1.91 ± 1.35	0.081	↑ Higher scores indicate greater likelihood of cognitive impairment [12]
	Cognitive Reserve Scale (CRS)	140.44 ± 26.25	132.18 ± 32.88	0.324	↓ Lower scores indicate lower cognitive reserve [14]
Psychological/ Functional	Geriatric Depression Scale (GDS) (0–15)	3.48 ± 3.37	2.32 ± 2.41	0.082	↑ Higher scores indicate more severe depressive symptoms [45]
	ECog	51.56 ± 14.71	50.24 ± 11.42	0.674	↑ Higher scores indicate greater functional cognitive difficulties [50]
	ADLQ basic	1.42 ± 4.17	0.82 ± 2.29	0.434	↑ Higher scores indicate greater basic functional impairment [10]
	ADLQ instrumental	9.17 ± 11.41	8.06 ± 7.64	0.649	↑ Higher scores indicate greater instrumental functional impairment [10]
	ADLQ advanced	21.97 ± 18.32	27.65 ± 21.35	0.517	↑ Higher scores indicate greater advanced functional impairment [10]

* Indicates the age range (max-min).

The possibility that the variables in the metadata could explain differences in the abundance of specific bacterial genera was also addressed. The results reveal that the participants’ sex is the only variable that can induce statistically significant changes in the abundance of bacterial genera, with *Mogibacterium* found in higher abundance in males compared to female participants (Fig 1D). Our combined results indicate that although the gut microbiome of the GERO cohort is predominantly composed of the phyla Firmicutes and Bacteroidota, its composition is influenced by anthropometric factors, performance on the ADLQ and ECog tests, as well as depressive symptoms scores.

### 3.2 The gut microbiomes of the GERO cohort show several pathways for the synthesis and degradation of neuroactive molecules

**Fig 2:**
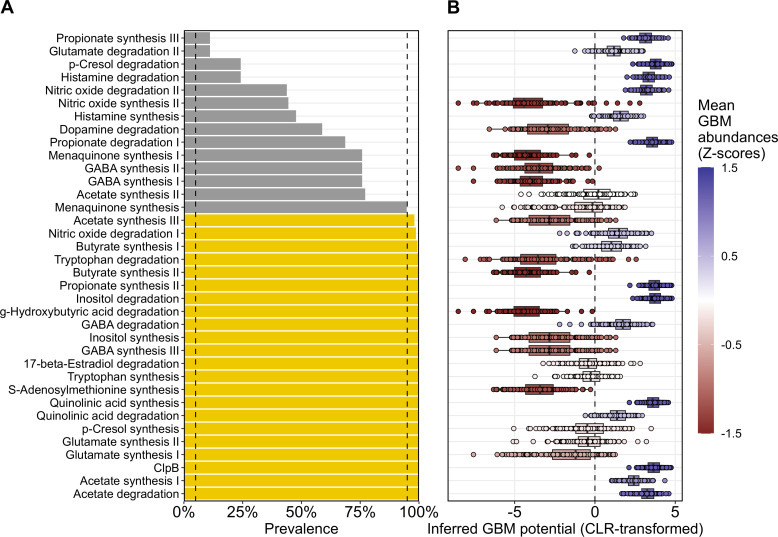
Characterization of the inferred neuroactive potential of the gut microbiome of the GERO cohort. (A) GBMs Prevalence, defined as the proportion of the participants among the total, in which each GBM was inferred to be fully present. While yellow indicates GBMs with a prevalence above 90%, grey shows GBMs with prevalence ranging from 5% to 90%. (B) CLR-transformed abundances of each GBM. Each dot represents one participant. Colours represent Z-scores calculated using the means of the CLR-transformed abundances for all GBMs, with red gradients indicating Z-scores with low abundance (values below 0), and blue gradients indicating Z-scores with high abundance (values above 0).

The inferred genomic content from 16S rRNA data was used to estimate the abundance of different pathways involved in the synthesis and degradation of neuroactive molecules, the GBMs [29]. Functional analysis revealed the presence of 36 different pathways, representing approximately 64% of the described GBMs [30]. This ratio indicates the existence of different pathways through which the gut microbiomes of the GERO cohort may modulate nervous system function. Additionally, the prevalence of each GBM in the cohort was determined as the percentage of participants in which each step of the GBM was detected as present (Fig 2A). Interestingly, while no GBM was found in less than 5% of the participants, a threshold used to identify rare GBMs, 61% of the GBMs were detected in over 90% of participants, and therefore termed ubiquitous. Particularly, 6 of the 9 GBMs involved in SCFA metabolism (66%) were found in at least 90% of the participants. Additionally, all four GBMs involved in the metabolism of tryptophan and its catabolites were detected in more than 90% of participants. However, pathways involved in the synthesis of important neuroactive molecules such as propionate (a SCFA), and glutamate, the precursor of the neurotransmitter GABA, were present in fewer than 25% of participants. This highlights distinct microbial-neural communication pathways across individuals, potentially providing novel venues for personalised therapeutic and or life-style interventions.

Additionally, the total abundance of each GBM was calculated for every participant by pooling the metabolic potential of all bacteria in their gut microbiome (Fig 2B). Interestingly, GBMs associated with propionate synthesis, inositol degradation, quinolinic acid synthesis, ClpB, and acetate synthesis and degradation were found in high abundance and prevalence. This may suggest that these bacterial pathways are associated with gut-brain axis related processes in this cohort. On the other hand, GBMs like acetate synthesis, tryptophan degradation, butyrate synthesis, GABA synthesis and degradation, inositol synthesis, and S-adenosylmethionine synthesis were found to be highly prevalent across the cohort but in lower abundances. Conversely, GBMs like propionate synthesis, along with p-cresol, histamine, nitric oxide, and dopamine degradation were found at low prevalence but at high abundances. The high abundance despite their low prevalence may reveal unique neuroactive pathways at the individual level. Our findings demonstrate that the abundance and prevalence of GBMs are not correlated, emphasising the need to consider both aspects when evaluating the neuroactive potential of a microbiome.

### 3.3 Anthropometric variables and ADLQ scores are associated with changes in the neuroactive potential of the gut bacteria

**Fig 3:**
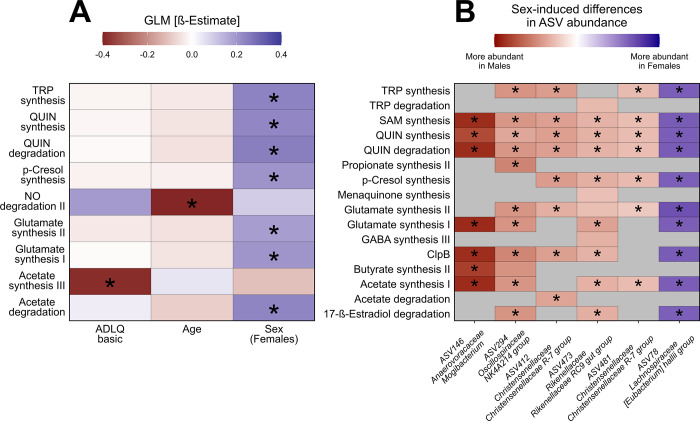
Age, Sex and ADLQ basic test score associated with changes in the neuroactive potential of the gut microbiome. (A) Variations in the overall neuroactive potential of the participant’s gut microbiome associated with changes in their covariates. A higher intensity of blue indicates either a higher abundance of the GBMs as age or ADQL test scores increases, and in female participants. Conversely, a more intense red indicates a lower GMB abundance under the same conditions. (B) Sex differences also contributed to statistically significant differences in specific GBMs from six different bacteria. Higher intensities of blue indicate a higher CLR-transformed abundance of the GBM of the specific bacteria in female participants. A more intense red indicates higher CLR-transformed abundances of the GBM in male participants. Grey areas indicate the absence of the GBM in the inferred bacterial genome. Both panels use colour intensity to represent the values of the β estimate in the GLM. Benjamini-Hochberg adjusted p values (q < 0.1) were used for determining significant differences, which are depicted with “*” in the figure panels. Abbreviations: TRP (Tryptophan), QUIN (Quinolinic Acid), NO (Nitric Oxide), SAM (S-Adenosylmethionine).

Since we showed that the taxonomic composition of the gut microbiome is influenced by anthropometric and mental variables, we hypothesized that those variables could also modulate the neuroactive potential of the gut microbiome. It was found that the participant scores on the ADLQ basic test, as well as participants’ age and sex have an effect in the abundance of 9 GBMs. Female participants showed a higher potential to synthesise tryptophan, quinolinic acid, p-cresol and glutamate, and also a higher potential to degrade quinolinic acid and acetate. Additionally, our results suggest that as participants’ age increases, their gut microbiome shows a reduced potential to degrade nitric oxide. Our results also showed a decreased potential to synthesise acetate of participants who score higher in the ADLQ basic tests (Fig 3A).

Additionally, the effects of the same covariates on the neuroactive potential of specific bacteria were tested, but only the sex of the participants was found to induce statistically significant changes. Sex has a significant effect on the neuroactive potential of 6 different ASVs, spanning 5 different genera, altering a total of 16 different GBMs. Results suggest that all the GBMs detected in the genera *Anaerovoracaceae Mogibacterium*, *Christensenellaceae Christensenellaceae R-7 group,* as well as some GBMs detected within the genera *Oscillospiraceae NK4A241* and *Rikenellaceae Rikenellaceae RC9 gut group* are more abundant in male participants. Conversely, all GBMs detected within the *Lachnospiraceae Eubacterium hallii group* were more abundant in female participants (Fig 3B). Together, our results indicate that anthropometric variables such as age and sex, along with the ADLQ basic test scores are associated with changes in the potential to synthesise and degrade neuroactive compounds relevant to the functioning of the CNS.

### 3.4 Depressive symptoms score and mental test performance are associated with neuroactive potential in the gut microbiome

**Fig 4:**
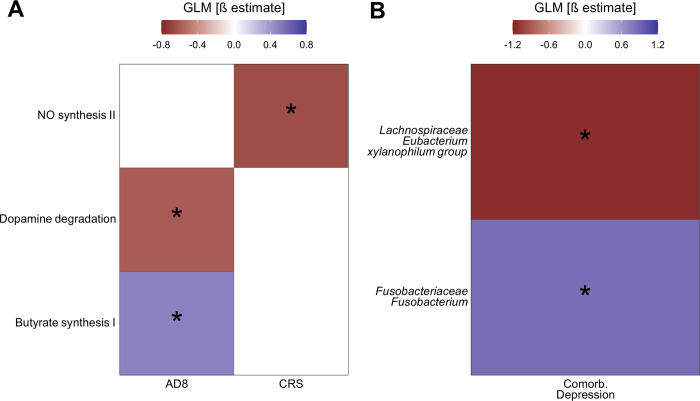
Mental test scores are associated with changes in the neuroactive potential of the gut microbiome and depressive symptoms are linked to specific gut bacterial taxa. (A) Associations between scores in the mental tests AD8 and CRS and the neuroactive potential of the gut microbiome. While blue represents a higher GBM abundance in participants with higher scores in the tests, red represents the opposite. (B) Associations between the abundance of genera and the depressive symptoms of the participant. “Comorb. Depression” denotes patients with depressive symptoms as a comorbidity. Blue represents a higher abundance of the bacteria in depressive participants, and red represents the opposite. In both panels, colours represent the β estimate of the GLM. Benjamini-Hochberg adjusted p values (q < 0.1) were used for determining significant differences and are depicted with “*” in the figure panels.

Several mental tests are used in older adults to evaluate cognitive function and assist in diagnosing neurological and neurodegenerative diseases. It was hypothesised that changes in the gut microbiome composition and its neuroactive potential would be associated with test performance. Our results suggest that higher scores in the AD8 tests are associated with lower gut microbiome potential to degrade dopamine and with higher potential to synthesise butyrate (Fig 4A). On the other hand, higher scores of CRS are associated with a decreased potential of the gut microbiome to synthesise nitric oxide (Fig 4A). Although functional changes in the gut microbiome were associated with participant score in two mental tests, no statistically significant associations were found between the abundance of any gut-bacterial taxa and test scores.

Additionally, it was evaluated whether participants self-reporting depression showed distinct gut microbiome compositions or different neuroactive potentials when compared to the rest of the cohort. Results indicate that participants with self-reported depression harbour a decreased abundance of the genera *Lachnospiraceae Eubacterium xylanophilum group*, and a higher abundance of the genera *Fusobacteriaceae Fusobacterium* (Fig 4B). However, no statistically significant changes were found in the neuroactive potential of their gut microbiomes. Together, these results link AD8 and CRS test scores to specific changes in the metabolism of nitric oxide, dopamine, and butyrate, which are not associated with the abundance of specific bacteria. On the other hand, in participants with depressive symptoms, two bacteria from the gut microbiota were found to be associated with this comorbidity.

## 4. Discussion

There is a growing emphasis on the potential of the microbiome to be a hallmark of aging in health and disease [26]. To the best of our knowledge, this is the first time the composition and neuroactive potential of the gut microbiomes of a population of elderly South Americans with cognitive complaints is associated with their performance in tests evaluating participants’ mental health and functional decline. Specifically, we found changes in the abundance of two bacterial genera in participants with self-reported depression. Additionally, changes in the abundance of functional pathways involved in the metabolism of neuroactive compounds were associated with changes in participants’ score in the AD8 and CRS tests. Therefore, our findings provide new insights into the role of intestinal microbiota in the cognitive decline and mental health of an ageing population with cognitive complaints from the global south.

The gut microbiome of the cohort shows a taxonomic composition (Fig 1A) similar to other human populations, dominated by Firmicutes and Bacteroidetes, followed by less predominant phyla such as Actinobacteria, Proteobacteria and Verrucomicrobiota [62,63]. Previous research on a younger and smaller group of Chilean participants showed similar findings [64]. One noticeable difference however pertains to the genera *Akkermansia*. Here, as well as in the younger cohort of Chileans, *Akkermansia* was the only genera within the phylum Verrucomicrobiota (previously known as Verrucomicrobia) detected. However, while it was previously identified as part of the core Chilean microbiota (defined in that article as genera that were present in every individual of the cohort), in the GERO cohort, sequences assigned to *Akkermansia* were found only in 66.0% of the participants. Although evidence has shown that *Akkermansia* is more abundant among the healthy aged populations [26,28], the evidence regarding changes in its prevalence is less convincing. Notably, *Akkermansia muciniphila* has been consistently reported as enriched in long-lived individuals, including semi-supercentenarians and centenarians, where its abundance is considered a microbial signature of healthy ageing [65]. As a key mucin degrader and SCFA producer, *Akkermansia* contributes to gut barrier integrity and immunometabolic homeostasis, mechanisms that may influence gut–brain communication [66]. Therefore, its lower prevalence in our cohort of older adults with cognitive complaints may reflect early deviations from microbiome profiles typically associated with healthier ageing trajectories.

Our analysis confirms that sex, age, and BMI significantly, albeit moderately, influence the taxonomic composition of the gut microbiome (Fig 1B,1C), similar to other European cohorts [30]. Additionally, it was previously shown that non-anthropometric variables like the quality of life of a participant, assessed by using the RAND 36-item survey, could explain up to 1.48% of the overall taxonomic changes in the gut microbiome [30]. Interestingly, we report a close percentage (1.56%) of explained taxonomic variation using the three ADLQ questionnaires (Fig 1B), which evaluate participants’ functional capacity. Although developed with a different scope, ADLQ assesses participants’ quality of life, physical functioning and mental health. Therefore, our results using ADLQ align with previous observations of the effects of the participants’ quality of life on shaping the gut microbiome composition [30].

Interestingly, it has been reported that men perform worse than women in memory, mental speed and global cognitive function in an elderly cohort [67], and that patients with cognitive complaints showed an increased abundance of *Mogibacterium* [68,69]. Our results showed a higher abundance of the genus *Mogibacterium* among male participants within a cohort of participants reporting cognitive complaints (Fig 1D), supporting the observations of the aforementioned works. This may suggest a role of the gut microbiome regarding sex differences in cognitive function and healthy ageing in humans.

The prevalence of GBMs within the gut microbiome of the participants was assessed. Our results align with a previous observation [30], as we found that all the GBMs previously defined as ubiquitous within bacterial genomes were present in more than 90% of the cohort participants, and GBMs previously defined as rare were also identified as less prevalent in the gut microbiome of the participants (Fig 2A). Interestingly, our results suggest that the extent of the prevalence of a GBM is not indicative of how abundant that GBM is within the bacterial community (Fig 2B). However, the biological interpretation of these observations remains elusive, and more research is warranted.

Our results suggest a sex-related pattern in the predicted microbial potential to metabolize tryptophan (TRP) and quinolinic acid (QUIN), rather than a consistent or measured effect (Fig 3A). On the one hand, TRP has been implicated in the modulation of the gut barrier permeability, either directly [70], or through catabolites generated by the metabolic activity of the gut microbiome [71]. Moreover, subsequent changes in permeability could in turn alter the exchange of TRP, QUIN and other metabolites between the gut lumen and the bloodstream, from where they can reach the brain. For instance, TRP can reach the brain after its transportation through carriers [72]. TRP is also the essential precursor for serotonin synthesis, yet it cannot be produced endogenously and must be obtained exclusively through dietary intake [73]. This dependence on dietary TRP highlights the importance of gut microbial metabolism in regulating its availability for neurotransmitter pathways, and suggests that alterations in TRP-related microbial functions may have downstream effects on serotonergic signaling in ageing populations [74]. Conversely, QUIN, a molecule with neurotoxic properties, has been associated with neurological disturbances that can become more prevalent as individuals grow older [75], has been shown to accumulate in animal models as they age, particularly in the context of inflammatory neurological diseases [76,77]. Additionally, an increase in QUIN level in blood has been linked to increased prevalence of Alzheimer’s disease [78,79], which is more prevalent in women than men [80,81]. Moreover, in susceptible individuals, reduced plasma concentrations of TRP have been associated with mood alterations, including an increased risk of depressive symptoms [74]. Such findings emphasize the relevance of TRP availability and metabolism in maintaining emotional stability, particularly in ageing populations [74]. It is important to note that the neuroactive potential described here is based on predicted functional pathways inferred from 16S rRNA data, and therefore represents functional capacity rather than directly measured metabolite levels.

Our results also suggested that female participants showed an increased potential to synthesise p-cresol (Fig 3A), a molecule whose abundance in the stool of aged humans has been linked with increased frailty in the ELDERMET cohort [82]. Additionally, another study integrating three independent cohorts showed that p-cresol and its associated metabolites were found in higher abundances in aged participants, where an effect of the sex of the participant on the abundance of the molecule was also described [17]. This aligns with our observations and supports the need for further investigation into sex-related microbial differences.

Our studies reveal that the gut microbiomes of female participants exhibited an increased potential to degrade acetate and that a reduced potential of the gut microbiome to synthesise acetate is associated with higher scores in the ADLQ basic test, an indicator of a higher level of dependence (Fig 3A). Acetate levels decrease in aged mice, and its supplementation improved their health span along ageing [83]. Moreover, other work shows how the removal of acetate-producing bacteria from the gut of aged mice is associated with learning and memory complaints, along with synaptophysin reduction in the hippocampus [84], a protein important for synapse formation within the hippocampus [85]. Interestingly, mice synaptophysin levels could be restored after acetate supplementation [84]. Furthermore, in individuals with depressive symptoms, plasma acetate levels have been reported to correlate positively with symptom severity, whereas butyrate and propionate show negative correlations [86]. These patterns are consistent with the notion that shifts in SCFA profiles may contribute to mood dysregulation through metabolic and inflammatory pathways. Similarly, a study in Polish women with depression reported reduced fecal acetate and propionate levels, suggesting that maintaining a balanced SCFA profile may be important for supporting mental health [34]. While additional work is necessary to understand the translational implications of these findings, the evidence highlights the relevance of our results as it emphasises sex-driven differences in the metabolism of a molecule crucial to healthy ageing in animal models.

Additionally, we found that as the participants’ age increases, the gut microbiome potential to degrade NO decreases (Fig 3A). This may suggest an accumulation of NO in the gut environment, which has been linked to increased gut permeability [87], a condition usually associated with health challenges in older individuals [25,88]. Elevated oxidative stress has also been linked to depressive symptoms [89], suggesting that age-related impairments in nitric oxide metabolism may contribute to broader biochemical imbalances associated with mood vulnerability in older adults. Nevertheless, more studies are needed to further investigate the role of the gut microbiome in aged cohorts from the global south. In that respect, other experimental designs such as cross-sectional or longitudinal studies may unveil further insights into the role of the gut microbiome in healthy ageing in South American subjects.

We also reported associations between the participants’ performance on the AD8 and CRS tests, and the neuroactive potential of their gut microbiome, which are the first of their kind described in populations outside the global north. As mentioned before, the AD8 test is an informant-based questionnaire aimed at early detection of cognitive changes indicative of dementia or cognitive decline associated with Alzheimer’s disease [12]. Interestingly, a meta-analysis of cross-sectional studies suggests that increased inflammation is associated with Alzheimer’s disease and mild cognitive impairment among the elderly [90]. Noteworthily, butyrate [91] and dopamine [92] in the gut have been implicated in the modulation of systemic inflammation, which aligns with our results showing associations between the scores in AD8 and both metabolites (Fig 4A). This suggests a potential indirect mechanism by which the gut microbiome may influence the cognitive performance of the elderly through the modulation of host systemic inflammation, a concept supported by existing research linking the gut microbiome to the modulation of this process [26].

Additionally, we observed that higher scores in the CRS test were associated with a reduced potential of the gut microbiome to degrade NO (Fig 4A). Importantly, NO has been implicated in regulating gut permeability [87], and modulating the synthesis of brain-derived neurotrophic factor (BDNF) in the brain [93]. BDNF plays an important role in neuronal survival, growth, and neural plasticity [94]. Interestingly, neural plasticity is a key mechanism in cognitive reserve and a pivotal characteristic that accounts for individual differences in susceptibility to age-related brain changes or Alzheimer’s disease-related pathology [95]. However, further research should explore how alterations in intestinal NO levels might affect the production of BDNF in the brain, and its potential to cross the brain barrier.

Additionally, we found that participants with self-reported depressive symptoms exhibited a lower abundance of the genera *Lachnospiraceae Eubacterium xylanophilum group*, and a higher abundance of the genera *Fusobacteriaceae fusobacterium* (Fig 4B). Interestingly, there is still a lack of consensus regarding the association of specific bacteria and depression symptoms. While higher abundance of *fusobacterium* in patients with depressive symptoms has been shown [96], the opposite association has also been reported [97]. However, in other mental health conditions such as anxiety disorders, increased abundance of *Fusobacterium* has also been reported, suggesting that its role may vary across different psychological phenotypes and may not be specific to depressive symptoms alone [98]. To our knowledge, associations involving the *Lachnospiraceae Eubacterium xylanophilum* group and depression have been rarely explored in the literature, and the signal observed here should be interpreted cautiously [99]. In addition, members of the genera *Clostridium* and *Eubacterium* are major contributors to the transformation of primary into secondary bile acids, and have been linked to cholesterol metabolism as well as the regulation of neurotransmitter pathways [100]. Through these mechanisms, alterations in their abundance may influence neurotransmitter receptor function and contribute to mood- and cognition-related disturbances. Noteworthy, associations between abundances of bacteria and symptoms or self-reported illnesses must be taken with care, especially when the gut microbiome is profiled with marker genes [101].

It is worth noting that this study is not without its limitations. Firstly, the absence of a group of elderly individuals without cognitive dysfunction used as a control makes this an observational study. Additionally, it remains to be understood to what degree the observed mood effects are specific to cognitive dysfunction and whether they can be generalized to a cognitive decline-free population. However, a closer inspection of experimental designs used in clinical studies have shown that the magnitude of the effects of treatment in well-designed observational studies don’t systematically differ with those reported in experimental designs of higher levels of evidence [102]. Additionally, the plethora of lifestyle patterns known to modulate the composition of the gut microbiome, such as diet, physical activity, accessibility to green areas and exposure to xenobiotics and environmental pollutants couldn’t be totally accounted for in this study. Future investigations, especially emerging from the Global South, should address these factors [103,104]. Finally, besides the aspects already discussed, future studies conducted in similar populations should also consider the temporal dynamics of the human gut microbiome for a personalised understanding of health- and disease-associated microbiome patterns [105]. Moreover, the cross-sectional ature of this study, limits its potential to unveil causal mechanisms. Further studies must include larger samples to increase the detection of subtler differences. Finally, future studies relying on metagenomic analysis integrated with other omics approaches are required to better assess the functional relevance of the pathways here highlighted. Additionally, the GERO cohort analyzed in this study includes only older adults with cognitive complaints and does not incorporate cognitively healthy controls. Nonetheless, our research group has recruited additional, independent participant groups, including healthy older adults and individuals with neurodegenerative diseases. as part of the broader GERO research program. These groups were not included in the present analysis but will be incorporated in future comparative studies.

In conclusion, this study has characterised the gut microbiome composition and neuroactive potential of a population of elderly Chileans, and described its associations with participants’ anthropometric variables, cognitive measures, and depressive symptoms. In particular, the inferred microbial potential to metabolise TRP, SCFAs, p-cresol, glutamate, and NO varied with age, sex, and performance on mental tests such as the basic ADLQ, AD8, and CRS. Moreover, two bacterial genera, *Lachnospiraceae Eubacterium xylanophilum group* and *Fusobacteriaceae Fusobacterium,* showed differences in abundance among participants who self-reported depression. Notably, the findings suggest sex-associated differences in inferred neuroactive potential of gut microbiota in the elderly with cognitive complaints varies between men and women, which may help guide future investigations into possible sex-specific patterns during ageing. However, further studies are required to clarify the biological relevance of these associations and to better understand the role of the gut microbiome in the ageing brain.

## Supporting information

S1 Table daa_supp_table. (CSV)
